# Challenges and Opportunities from Targeting Inflammatory Responses to SARS-CoV-2 Infection: A Narrative Review

**DOI:** 10.3390/jcm9124021

**Published:** 2020-12-12

**Authors:** Vincenzo Lariccia, Simona Magi, Tiziano Serfilippi, Marwa Toujani, Santo Gratteri, Salvatore Amoroso

**Affiliations:** 1 Department of Biomedical Sciences and Public Health, School of Medicine, University “Politecnica delle Marche”, Via Tronto 10/A, 60126 Ancona, Italy; s.magi@staff.univpm.it (S.M.); s1065931@studenti.univpm.it (T.S.); s1072476@studenti.univpm.it (M.T.);; 2 Institute of Legal Medicine, University “Magna Graecia”, 88100 Catanzaro, Italy; gratteri@unicz.it

**Keywords:** anti-inflammatory drugs, cytokine storm, COVID-19, immunomodulation

## Abstract

The novel coronavirus disease 2019 (COVID-19) is a global pandemic that continues to sweep across the world, posing an urgent need for effective therapies and prevention of the spread of the severe acute respiratory syndrome related to coronavirus-2 (SARS-CoV-2). A major hypothesis that is currently guiding research and clinical care posits that an excessive and uncontrolled surge of pro-inflammatory cytokines (the so-called “cytokine storm”) drives morbidity and mortality in the most severe cases. In the overall efforts made to develop effective and safe therapies (including vaccines) for COVID-19, clinicians are thus repurposing ready-to-use drugs with direct or indirect anti-inflammatory and immunomodulatory activities. Speculatively, there are many opportunities and challenges in targeting immune/inflammatory processes in the evolving settings of COVID-19 disease because of the need to safely balance the fight against virus and aggressive inflammation versus the suppression of host immune defenses and the risk of additional harms in already compromised patients. To this end, many studies are globally underway to weigh the pros and cons of tailoring drugs used for inflammatory-driven conditions to COVID-19 patient care, and the next step will be to summarize the growing clinical trial experience into clean clinical practice. Based on the current evidence, anti-inflammatory drugs should be considered as complementary approaches to anti-viral drugs that need to be timely introduced in the management of COVID-19 according to disease severity. While drugs that target SARS-CoV-2 entry or replication are expected to confer the greatest benefits at the early stage of the infection, anti-inflammatory drugs would be more effective in limiting the inflammatory processes that drive the worsening of the disease.

## 1. Introduction

The severe acute respiratory syndrome related to coronavirus-2 (SARS-CoV-2), the infectious agent of the so-called coronavirus disease 2019 (COVID-19), is a novel coronavirus initially identified in Wuhan City, Hubei province in China, during December 2019, in patients who manifested an atypical pneumonia characterized by fever, dry cough and progressive dyspnea [1,2]. In just two more months, the virus spread all over the world and, in 11 March 2020, the World Health Organization (WHO) declared COVID-19 as pandemic. Since December 31, 2019, and as of 4 December 2020, 64,350,473 cases of COVID-19 (in accordance with the applied case definitions and testing strategies in the 220 affected countries) have been reported worldwide, including 1,494,668 deaths [3].

SARS-CoV-2 is a beta-coronavirus, like the severe acute respiratory syndrome coronavirus (SARS-CoV) and the Middle East respiratory syndrome coronavirus (MERS-CoV), both responsible of devastating outbreaks over the last 20 years [4]. It is an enveloped, positive-sense, single stranded RNA virus with a genome size of around 30 kb which encodes for 29 proteins involved in the processes of infection, replication and virion assembly [5]. The SARS-CoV-2 entry into the host cell is mediated by a virus surface-anchored spike protein (providing the typical “crown” appearance), which contains a receptor-binding domain (RBD) that specifically recognizes angiotensin-converting enzyme 2 (ACE2) as its receptor [6]. ACE2 is abundantly expressed on alveolar epithelial type II cells [7], which represent 83% of all ACE2-expressing cells, but its expression has been also described in nasal mucosa, upper respiratory tract, endothelium, heart, kidney, endothelium, and gut cells [7,8]. A process of endocytosis, promoted by membranes fusion, represents the main entry pathway of SARS-CoV-2 on human ACE2 expressing cells [9].

**Figure 1:**
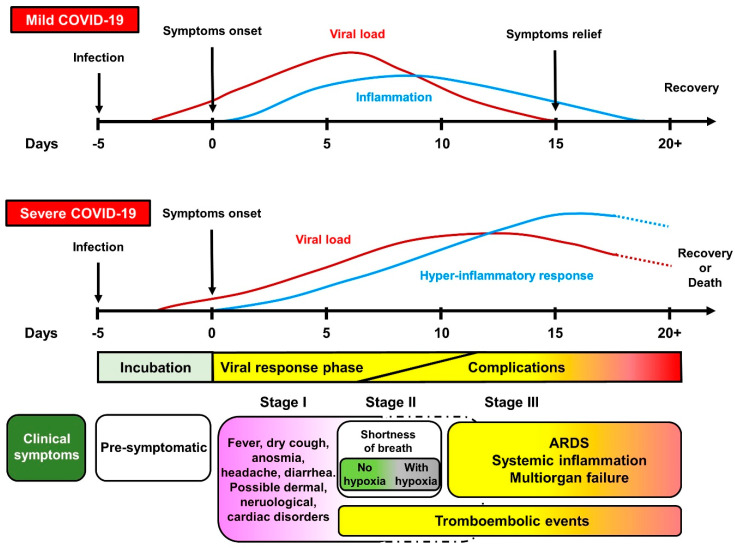
Spectrum and clinical stages of COVID-19. Clinical symptoms that correlate the disease course were scaled to the average days and time windows at which they typically occur. In general, the incubation period is on average 5 days from SARS-CoV-2 infection. Most people will experience mild to heavy symptoms during Stage I (which roughly correlate with peaks in viral load and inflammatory response) and no complications. Severe COVID-19 is instead characterized by a hyper-inflammatory response that incites disease progression toward Stage II and III, with acute respiratory distress syndrome (ARDS), multiorgan failure, and coagulation disorders as main causes of death.

A staged progression model of escalating and partially overlapping phases (Figure 1) has been proposed to guide the clinician in choosing the most appropriate therapy [10,11]. According to this model, the initial stage refers to the early infection period, when the virus multiplies and establishes residence in the host. At this stage, patients may or may not manifest rather non-specific symptoms (muscle pain, fever, headache, chills, sore throat, dry cough). The host–virus interaction then eventually incites an inflammatory response that can lead to respiratory problems and, in a minority of infected individuals, further drive the deterioration of patients’ clinical condition to acute respiratory distress syndrome (ARDS), systemic inflammation, thromboembolic manifestations (e.g., pulmonary embolism and disseminated intravascular coagulation) and multiorgan failure [12,13]. Patients experiencing these latter complications possess the worst prognosis.

Compelling evidence has shown that the severity of the disease is related to the levels of the pro-inflammatory cytokines and subsets of immune cells, both representing the main causes of tissue injury during SARS-CoV-2 response [14]. The excessive and uncontrolled release of pro-inflammatory cytokines results in the so-called “cytokine storm” which, in turn, can induce acute respiratory distress syndrome, respiratory and organ failure, potentially leading patients to death. SARS-CoV-2 infection may also lead to immune cells dysregulation, by affecting the subsets of T cells. Patients experiencing a severe disease course display a significantly low number of T lymphocytes, including both CD4+ and CD8+ subtypes and especially of NK cells. The number of regulatory T cells is also very low. In contrast, monocytes and macrophages are increased and represent the great majority of the inflammatory cells infiltrating the lungs, explaining the elevated levels of pro-inflammatory cytokines described above [15].

The majority of infected individuals (nearly 80 %) are asymptomatic, or present mild symptoms, conditions that have been ascribed to an efficient immune response able to adequately control the disease progress [16]. Notably, evidence so far available suggests that asymptomatic individuals can spread the disease to other people [17]. Approximately 15% of infected people presenting moderate to severe symptoms may develop both multiorgan and primary respiratory failure, requiring admission to an intensive unit care (IUC).

**Figure 2:**
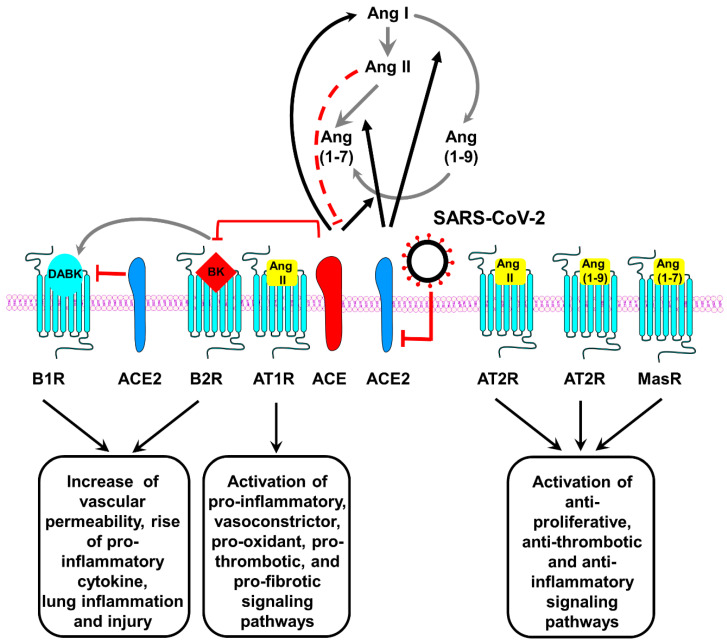
Role of the Renin–Angiotensin Systems in the pathobiology of SARS-CoV-2 infection. Black arrows indicate enzymatic activation; red lines represent inhibition or degradation/downregulation. Dashed red lines entail the involvement of other molecules (not depicted) to inhibit the molecule indicated at the end of the line. Gray arrows indicate conversion of one molecule into another. ACE, angiotensin converting enzyme; ACE2, angiotensin converting enzyme 2; Ang I, Angiotensin I; Ang II, Angiotensin II; Ang (1-7), Angiotensin 1-7; Ang (1-9), Angiotensin 1-9; AT1R, ATII type 1 receptor; AT2R, ATII type 2 receptor; B1R, B1 receptor; B2R, B2 receptor; BK, bradykinin; DABK, [des-Arg9]-BK; MasR, Mas receptor. Modified from [27].

The mechanisms underlying such a heterogeneous course are still puzzling. Trends in COVID-19 severity and death are increased by three epidemiological factors: older age, being male and previous pathological conditions (e.g., obesity, diabetes mellitus, hypertension, and coronary heart disease) [18,19,20]. The degree of ACE2 expression has also the potential of influencing the course of COVID-19: although ACE2 is critically required for SARS-CoV-2 binding, internalization and viral infection, its downregulation may facilitate hydrostatic edema formation, hypoxia-induced pulmonary vasoconstriction and apoptosis of alveolar epithelial cells (AECs) because of an excess of the pro-inflammatory mediator angiotensin-II (Ang II) that, in this scenario, would not be efficiently converted by ACE2 in angiotensin (1-7) peptide (Ang 1-7), which instead acts as vasodilator, protects against acute lung injury and promotes AEC survival (Figure 2) [21,22,23,24]. Another negative consequence of ACE2 depletion is related to the extended half-life of des-Arg(9)-bradykinin (BK) (a bioactive metabolite of BK lacking the arginine residue at position 9). In particular, a reduction in ACE2 activity will also compromise the degradative pathway for des-Arg(9)-(BK) that may further promote the inflammatory status and lung injury (Figure 2) [21,22,25,26].

Very recently, evidence provided by an international consortium suggests that life-threating forms of COVID-19 can be traced to genetic or immunological defects that prevent effective immune responses driven by type I interferon [28,29].

In a worldwide rush to tackle the coronavirus pandemic, many efforts are being currently paid to diagnose, treat and prevent spreading infections from the SARS-CoV-2 virus. Although new insights continuously refine/integrate the above-mentioned pathological paradigm, there is presently no molecule in the pipeline that will end up as “wonder” drug against COVID-19 in the near future [30,31,32]. To date, remdesivir is the only antiviral drug that was granted a conditional marketing authorization in the European Union (3 July 2020) with the specific therapeutic indication to treat COVID-19 in adults and adolescents (aged 12 years and older with body weight at least 40 kg) with pneumonia requiring supplemental oxygen [33]. However, shortly after the publication of the final report from a large-scale trial that indicated benefits of using remdesivir in hospitalized COVID-19 patients [34], on 15 October 2020 the WHO announced interim results from the SOLIDARITY trial (a randomized study conducted in 405 hospitals across 30 countries on 11,266 patients, with 2750 given remdesivir) suggesting little or no effect on hospitalized COVID-19 treated with remdesivir [35]. Another chapter in proof in the chronicles of therapy regards vaccines against the SARS-CoV-2. [32,36,37,38]. Even if we consider the flurry of recent vaccine announcements from pharmaceutical giants as reason to think positively about a return to normality [39,40,41], once a vaccine gets emergency approval, large-scale vaccination campaigns around the world should then follow (which are not simple tasks) and, inevitably, it will take some other time to study and verify the impact of vaccines on COVID-19 pandemic [42,43,44]. In the meantime, the use of several drugs developed or approved for other indications is in parallel evaluated as an opportunity to target and manage symptoms of COVID-19 and (eventually) co-existing medical conditions, as well as debated within the medical community and health authorities around the world [31,32]. Anti-inflammatory therapies are, for instance, being considered in multidisciplinary approaches to halt hyper-inflammatory responses that would otherwise deadly incite COVID-19 progression, and many efforts are being paid to test molecules like anti-cytokine biologicals and disease-modifying antirheumatic drugs (DMARDs) [45,46]. While there is a general consensus that drugs with anti-inflammatory/immunomodulatory effects can potentially disrupt the self-perpetrating loop that dictates the transition from mild to severe form of COVID-19 [47,48], we are still on the road to figuring out which therapeutic approaches are best according to patients’ conditions [31,49,50,51]. For instance, molecules that target specific inflammatory pathways would be clinically relevant only if they timely hit on major signals among the redundant drivers of inflammatory storm caused by SARS-CoV-2 infection. In addition, targeted anti-inflammatory therapies can either provoke compensatory adaptations able to bypass the original drug-inhibited signaling pathway, or even further limit the viral control when these therapies impair the first line of defense, the innate immunity, which may be compromised in severe and critical COVID-19 cases [52,53]. On the other hand, use of non-steroidal anti-inflammatory drugs (NSAIDs) and of corticosteroids (CSs) have been repeatedly debated during the SARS-CoV-2 pandemic, because conflicting evidence suggests benefits, harms or even neutral effects. In particular, the main concern is that, even if NSAIDs and CSs can potentially provide an effective brake to inflammatory drive, they can also add further burden to an already compromised patient [54,55,56,57].

Addressing all these relatively new issues is of paramount importance to guide therapy, considering the uncertainty in strategies used to quell cytokine storm in COVID-19 patients. Moreover, the growing spread of SARS-CoV-2 will inevitably hit individuals who are already on or would potentially use NSAIDs and CSs, which are extensively used worldwide with many therapeutic indications. In this short communication, we will discuss the potential pros and cons of drugs with anti-inflammatory properties in COVID-19.

## 2. Targeting Pro-Inflammatory Cytokine Signals in SARS-CoV-2 Infection

**Figure 3:**
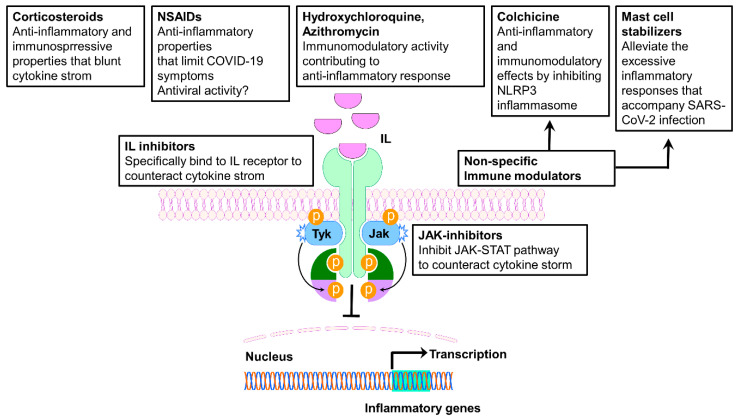
Schematic representation of the immunomodulatory and anti-inflammatory drugs’ site of action. IL, interleukin; IL-R, interleukin receptor; JAK, Janus kinase; JAK-STAT, Janus kinase-signal transducer and activator of transcription.

The elevated serum levels of pro-inflammatory cytokines observed in most patients with severe COVID-19 provided the rationale to target specific cytokines and their intracellular signaling, in order to positively influence the progression of the disease. Accordingly, the efficacy of immunomodulatory agents, such as monoclonal antibodies and small molecules, has rapidly been investigated into several clinical trials, and many agents are now being increasingly used in an off-label manner. Among them, anti-IL-6, anti-IL-1, and JAK/STAT (Janus kinases/signal transducers and activators of transcription) (Figure 3) inhibitors have been related to a positive outcome.

### 2.1. IL-6 Receptor Antagonists

IL-6 is one of the central cytokines produced in the context of the SARS-CoV-2-induced cytokine storm, which is observed as the inflammatory response fully progress in an uncontrolled way [58]. It has been shown that IL-6 can suppress the functions of T lymphocytes, dendritic cells and macrophages that are “in charge” to eliminate coronaviruses. Consequently, the ability of the immune system to clear such infections is severely impaired [58]. Thus, it is possible to assume that IL-6 overproduction can be induced with the specific aim to escape immune surveillance. In this context, abnormally high levels of IL-6 have been related to a poor outcome in COVID-19 patients with pneumonia and ARDS [59].

Tocilizumab is one of the most largely studied monoclonal antibody for COVID-19 therapy. This recombinant humanized anti-human IL-6 receptor monoclonal antibody specifically binds both membrane-bound and soluble IL-6 receptor, thereby inhibiting receptor activation and signal transduction [60]. Currently, tocilizumab is approved to treat a number of severe autoimmune diseases including rheumatoid arthritis (RA), giant cell arthritis, polyarticular juvenile idiopathic arthritis, and systemic juvenile idiopathic arthritis. In addition, tocilizumab is approved for the management of chimeric antigen receptor (CAR) T-cell-induced cytokine release syndrome (CRS) [33]. In reason of this specific application, tocilizumab has emerged as a promising treatment in severely affected patients since the early stages of the pandemic COVID-19. Despite the great interest around this drug, the actual benefits coming from its use are still under evaluation, since most of the results reported in the literature comes from retrospective/prospective observational studies, the majority of which converge on an improvement of the clinical outcomes. For instance, a single center study conducted in Brescia [61] prospectively analyzed a series of 100 consecutive patients admitted to the Spedali Civili University Hospital between 9 and 20 March 2020, with confirmed COVID-19 pneumonia and ARDS requiring ventilatory support. The study was aimed to determine whether intravenous administration of tocilizumab was associated with improved outcome. The results reported that response to tocilizumab was rapid, sustained, and associated with significant clinical improvement. The TESEO study, a large, multicenter, retrospective cohort study enrolling 544 patients, reports a significant reduction of the risk of invasive mechanical ventilation or death in patients with severe COVID-19 pneumonia after treatment with tocilizumab, administered either intravenously or subcutaneously [62]. A larger retrospective observational cohort study conducted in 13 hospitals within the Hackensack Meridian Health network (NJ, USA) has reported encouraging results as well. The study included 764 patients (aged ≥18 years), with laboratory-confirmed COVID-19, which were admitted in the ICU between 1 March and 22 April 2020 [63]. Among them, 210 (27%) received tocilizumab and 420 did not. Tocilizumab reduced the mortality associated to COVID-19, since among the overall patients who died (358, 57%), 49% died in the tocilizumab-treated group (102 from 210) and 61% in the untreated group (256 from 420). Other similar experiences come from different Chinese studies [64,65], lending support to the possible effectiveness of tocilizumab and paving the way for its use as a valid therapeutic option for the CRS of severely ill COVID-19 patients. It is worth mention here that in June 2020, a study evaluating the effect of the early administration of tocilizumab conducted in the USL-IRCCS of Reggio Emilia was discontinued for the lack of benefits deriving from tocilizumab administration. The beneficial effects of tocilizumab most probably rely on its ability to counteract the cytokine storm. Thus, it is reasonable to hypothesize that the drug can act while the cytokine storm is ongoing, but it is not able to prevent it. Before implementing tocilizumab as standard care, the above-mentioned results need to be confirmed by high-quality clinical trials, which will help to identify the population of patients who could benefit from this therapy [66].

Sarilumab is another monoclonal antibody binding the IL-6 receptor. The drug has been licensed for the treatment of adult patients, with moderately to severely active RA, who have had an inadequate response or intolerance to one or more disease-modifying antirheumatic drugs. Although sarilumab is supposed to act similarly to tocilizumab, the literature does not report convincing results on its use. In particular, on September the first, Sanofi announced the discontinuation of the phase III trial evaluating sarilumab in severe and critically ill patients hospitalized with COVID-19. The 420-patients randomized trial was conducted outside the U.S., specifically in Argentina, Brazil, Canada, Chile, France, Germany, Israel, Italy, Japan, Russia and Spain. Patients were distributed as follows: 86 patients in placebo, 161 in 200 mg, and 173 in 400 mg arms). The study did not meet neither its primary endpoint (time to improvement of 2 points or greater on a 7-point clinical scale) nor its key secondary endpoint (percentage of patients alive at day 29) [67] compared to placebo.

### 2.2. IL-1 Receptor Antagonists

Anakinra is a recombinant human IL-1 receptor antagonist that blocks the biologic activity of IL-1α and IL-1β by competitively inhibiting their binding to the interleukin-1 type I receptor. Anakinra is licensed for RA and cryopyrin associated periodic syndromes. The documented effectiveness of anakinra in treating the macrophage activation syndrome [68], which is characterized by a strong cytokine storm, suggested a possible role of this drug in counteracting CRS-associated symptoms in COVID-19 affected patients. At present, no published controlled clinical trials are available on the efficacy and safety of anakinra use in COVID-19. The main results come from observational studies and needs to be confirmed by controlled trials. A cohort study on patients with severe COVID-19 showed that anakinra decreased both the need for invasive mechanical ventilation in the intensive care unit, and mortality (25% vs. 73%, hazard ratio (HR) 0.22, 95% confidence interval (CI) 0.11–0.41, p < 0.0001) without serious side-effects [69]. A small case-series of 9 patients experiencing moderate to severe COVID-19 pneumonia report positive results in terms of both safety and efficacy deriving from anakinra administration [70]. Another retrospective case series analyzed 14 patients with SARS-CoV-2 characterized by acute hypoxic respiratory failure (AHRF) [71]. The patients received anakinra for a maximum of 19 days. Among patients taking anakinra, 7 initiated ≤36 h after onset of AHRF and did not require mechanical ventilation. All of them were discharged home. 4 patients started anakinra ≥4 days after onset of AHRF; they all required mechanical ventilation. Of those, 3 patients were extubated (2 discharged home, 1 remains hospitalized) and 1 died. All 3 patients who met criteria but did not receive anakinra required mechanical ventilation. Two were extubated (1 discharged and 1 remains hospitalized) and 1 remains on mechanical ventilation [71]. Although preliminary, these results suggest that an early administration of anakinra (within 36 h from the onset of AHRF) could be beneficial in preventing the need of mechanical ventilation. A phase II/III, randomized, open-label, parallel group, 3-arm, multicenter study is investigating the efficacy and safety of administrations of emapalumab (an anti-IFNγ monoclonal antibody) and anakinra, versus standard of care, in reducing hyper-inflammation and respiratory distress in patients with SARS-CoV-2 infection. The study started on 2 April 2020 and its completion is estimated for December 2020 (NCT04324021). The trial is evaluating the intravenous infusion of anakinra, while other noncomparative, open-label studies that are ongoing in Greece (NCT04356366, NCT04339712) and Belgium (NCT04330638) are testing the subcutaneous administration of the drug.

### 2.3. JAK/STAT Inhibitors

Another attractive approach to restrain an overwhelming cytokine storm is through JAK-STAT pathway inhibition, which interferes with the signaling of several cytokines at once. This approach may be an effective strategy that potentially overcome the limit of targeting a single cytokine, considering the pleiotropic and partially redundant functions associated with most cytokines. In particular, Janus kinase inhibitors (JAKis) block cytokine signaling by inhibiting the phosphorylation of activated cytokine receptors. When activated, the phosphorylated cytokine receptors recruit STAT transcription factors which modulate gene transcription [72].

Drugs included in the JAKi group include baricitinib, tofacitinib, perficitinib, filgotinib, and upadacatinib [73]. It has been argued that JAKis may not be useful in the early stages of infection with SARS-CoV-2, since the activity of interferons, which are often the major mediators of viral clearance in the body, are mediated via the JAK-STAT signaling pathway. JAKis have been proposed as a treatment in severe coronavirus infection with features akin to cytokine storm.

In addition to immunologically relevant targets, selected agents may also impair viral entry. Upon binding its cell surface receptor (ACE2), SARS-CoV-2 viral particles gain entry into the cell by clathrin-mediated endocytosis, a process which requires a number of adaptor proteins and kinases, including AP2-associated protein kinase 1 (AAK1) and G-associated kinase (GAK) [74]. AAK1-dependent endocytosis is utilized by many viruses, and targeted agents that impair its activity inhibit viral entry [75,76]. Baricitinib, a JAK1/JAK2/TYK2 inhibitor, was observed to inhibit AAK1 (and related kinases) at clinically achievable concentrations (IC50 < 50 nM) [74,77]. This activity may be relatively specific to baricitinib, as inhibition by alternative JAK inhibitors (ruxolitinib and fedratinib) was less significant, with IC50s in cell-based assays approaching 1 μM [77].

Though some preliminary results were promising [49], there is currently need for randomized controlled trials to assess the safety and efficacy of JAKis. Clinical trials are currently being conducted to evaluate the value of JAKis as COVID-19 treatment [49].

## 3. NSAID Use During COVID-19 Pandemic: Pros and Cons

NSAIDs constitute a large and chemically heterogeneous group of compounds, with analgesic, anti-inflammatory and anti-pyretic properties that provide clinical benefits for both acute and chronic pathological conditions [78]. The inhibition of cyclooxygenase enzymes (COX-1 and COX-2) is classically proposed as the molecular mechanism that mostly accounts for their pharmacological properties, even though several effects not strictly related to their action on COX enzymes have been documented over the years [79]. Whether and how COX-dependent and COX-independent activities of NSAID provide benefits over harms are still at the root of an ongoing debate [56,57,80,81,82,83,84]. In particular, discussion sparked in March 2020 when the French authorities warned against using anti-inflammatory medications such as ibuprofen [83]. The bases for that warning mostly relied on anecdotal reports and non-peer-reviewed expert opinions, which went on to speculate that patients may be more vulnerable to SARS-CoV-2 infection if take drugs with proposed ability to increase ACE-2 expression, such as ACE inhibitors, Ang II receptor blockers and ibuprofen [57,84]. Soon after, numerous other global authorities urged prudence and several reports stressed the need for further evidence to get a clearer picture [85,86,87,88,89,90]. To this end, four registered trials are currently investigating the impact of ibuprofen (NCT04383899; NCT04334629; NCT04382768) and of naproxen (NCT04325633) on COVID-19 disease.

Even though therapy with NSAIDs promotes ACE-2 expression, theoretically providing more docking site for SARS-CoV-2, ACE-2 upregulation might also limit the severity of COVID-19 according to the mechanistic background depicted in the introduction [82,83,84]. In addition, even if NSAIDs are not considered as antiviral agents, provocative evidence not yet verified by clinical scrutiny suggests antiviral properties for NSAID molecules, including ibuprofen, indomethacin and acetylsalicylic acid (ASA) [84,91,92,93]. As far as ASA is concerned, the use of this drug in adult COVID-19 patients may offer two additional benefits: the antithrombotic effect and the production of the so-called aspirin-triggered anti-inflammatory mediators. In particular, ASA possesses the unique ability among members of NSAID class to irreversibly inhibit COX-1 in platelets and megakaryocytes, thereby limiting the synthesis of thromboxane A_2_, a potent platelet activator and vasoconstrictor [94]. ASA-dependent inhibition of platelet aggregation translates into clinical benefits since its use prevent arterial thromboembolic events and reduce venous thromboembolism, which have a high incidence rate and detrimental impact on prognosis of hospitalized COVID-19 patients [92,93]. In addition, acetylation of COX-2 by ASA modifies catalytic activity so that polyunsaturated fatty acids are hereafter converted in precursors for the synthesis of the epimeric forms of lipoxins and resolvins [95,96,97]. These latter are members of specialized pro-resolving mediators (SPMs) composed by lipidic molecules that can block inflammatory cell recruitment, inhibit cytokine release, and decrease vascular permeability [98,99].

Although at the present there is no specific reason to advise against all use of NSAIDs in patients with COVID-19, when NSAIDs are indicated, significant COVID-19-related clinical issues remain. In severe cases of COVID-19, ARDS is often associated with the development of a cytokine release syndrome. In this framework, a NSAID-dependent COX inhibition may reduce the recruitment of polymorphonuclear cells (in particular neutrophils) that during the course of inflammation eventually change the profile of lipid mediators produced toward synthesis of SPM metabolites [85,96,99,100,101]. Another potential issue to consider is that the anti-inflammatory and antipyretic effects of NSAIDs and acetaminophen may mask the early symptoms of infection and thus delay diagnosis and rapid management of the affected patients [56,57]. Administration of ASA to young COVID-19 patients may be similarly problematic: short-term use of ASA in children with either bacterial or viral infections can potentially lead to rare but life-threating complication called Reye’s syndrome [92]. Finally, severe forms of COVID-19 are strongly associated with risk of renal injury, myocardial events, and thrombotic complications, any of which may be exacerbated by the known risk profiles of various NSAIDs [79].

## 4. CSs Use during COVID-19 Pandemic: Pros and Cons

The term corticosteroids (CSs) is used clinically to describe synthetic derivates of hormones that are normally produced by the adrenal glands, which possess a steroid molecule that has been modified to intensify the glucocorticoid over mineralocorticoid activity. As such, CSs exhibit multiple glucocorticoid-type actions, including anti-inflammatory and immunosuppressive activities that rely on genomic and nongenomic mechanisms [102] and affect nearly all cells that participate in immunity and inflammation. In the light of these properties, systemic CSs have been empirically used to manage the cytokine storm in COVID-19 patients and mitigate its most severe manifestations such as ARDS, systemic organ failure and death [103,104]. In the early days of this pandemic, guidance regarding CSs was mixed: on the one hand, data collected during previous coronavirus outbreaks that share many clinical characteristics with COVID-19, SARS and MERS, were inconclusive about efficacy and safety of CSs [66,105,106,107]; on the other hand, the initial clinical experience on adjunctive therapy with CSs, which was essentially documented by case reports, observational and small (and often single-center) studies, similarly emphasized the need for large scale randomized trials to properly assess CSs on outcomes according to severity of COVID-19 disease [51,108,109,110,111,112,113,114,115,116]. On 6 June 2020, breaking news from the RECOVERY (Randomized Evaluation of COVID-19 thERapY) trial based in the United Kingdom (NCT04381936) announced that participants who received 6 mg dexamethasone daily for 10 days saw improvements, and in particular the subsets of patients already on ventilators or who required non-invasive oxygen therapy [117]. Compared to “usual treatment alone”, the 28-days mortality rate was lower in dexamethasone-treated patients, and in particular in the subgroups of patients receiving supplemental oxygen or invasive mechanical ventilation, but there was no benefit in COVID-19 patients not requiring respiratory support [118]. A subsequent meta-analysis that pooled data from 7 RCTs also highlighted beneficial signals on 28-day all-cause mortality from systemic use of CSs in hospitalized, critically ill patients with COVID-19 [119], suggesting that the benefit seen in these trials is a general class effect of CSs [104]. Overall, there is currently a reasonable ground for short-term application of low-to-moderate dose of dexamethasone (or equivalent) to COVID-19 patients with hypoxemia in peri-intensive care settings, when subjects may be entering a cytokine storm, rather than in less severe cases that can be managed with other (i.e., antiviral) drugs and/or supportive care. Benefits from CS therapy may be significantly higher when active viral replication is playing a secondary role for disease progression [118], and most probably rely on the following responses: suppression of aggressive surge of inflammatory cytokines, limitation of pro-inflammatory activities (including reduction of vascular leakage and tissue edema that would otherwise promote ARDS and organ injury), and on induction of proresolving lipid mediators [120,121]. However, there is still uncertainty in how to implement this therapeutic framework from “patients who meet trial eligibility criteria” to clinical practice. In particular, it is still a matter of debate how to optimize CS therapy according to illness severity, profile of biomarkers used to forecast evolution of the cytokine storm, and patient’s comorbidities (in particular the ones that may be exacerbated by CS). Similarly, it is also critical to have longer term follow-ups in order to identify delayed harms associated with CS use in COVID-19 patients. Timing and dosing regimens of CS use should be kept in consideration because these variables might influence outcomes during COVID-19 illness and after the recovery from SARS-CoV-2 infection. Immunosuppression by CSs would be of the greatest benefit within the hyperinflammatory phase of COVID-19, but also harmful during the initial host response to infection because CS-induced suppression of the innate immunity may increase the latent risk of superinfections and delay the clearance of the virus and, thereby, favor disease progression to severe stages. Prolonged use or moderate-to-high doses of CSs can, by themselves, either cause symptoms of iatrogenic hypercortisolism or provoke hypothalamic-pituitary-adrenal (HPA) axis suppression when CS withdrawal is carried without an appropriate dose tapering [54]. In the first case, any benefits from CS use would be likely outweighed by their known adverse effects [55], including hyperglycemia that is per se an independent predictor of mortality in hospitalized patients with COVID-19 [122,123]. In the second case, CS use may further compromise the HPA axis, which can be already dysfunctional in COVID-19 patients and increase the risk of adrenal insufficiency following CS discontinuation [54,55]. Finally, physicians should be also aware of possible interactions between CSs like dexamethasone and other drugs concomitantly used in COVID-19 patients, in particular those occurring at the level of cytochrome P450 (CYP) enzymes that may significantly influence the concentrations (and thereby the effects) of medications that are CYP substrates [54].

One last open question is worth to mention here: would the pre-morbid use or continued administration of inhaled corticosteroids (ICS) impact on clinical outcomes in COVID-19 patients? [124]. Unlike other viral respiratory pandemics (most recently the 2009 H1N1/Influenza A outbreak) or endemics (e.g., influenza), low prevalence of comorbid respiratory conditions (including asthma and chronic obstructive pulmonary disease (COPD)) has been repeatedly noted in patients with COVID-19 [125,126,127]. Among factors that may account for such epidemiological signal is that ICS, alone or in combination with bronchodilators, are widely used in the treatment of asthma and have a role in the management of some patients with COPD [124,125,126,127]. As such, ICS may exert protective effects against the respiratory symptoms associated with COVID-19 without necessarily limiting the systemic immune response to COVID-19 [124,125,126,127,128], but this hypothesis still needs to be verified in clinical trials [127]. Until more information is available, there is currently no evidence to support the COVID-19-due discontinuation of ICS in patients treated with these drugs [124,129].

## 5. Other Approaches to Quench Severe Inflammatory Reactions in COVID-19: Chances and Challenges for Therapy

The detrimental immuno-inflammatory reaction to SARS-CoV-2 is essentially a catalyst of multiple pathways, each of which can incite lung and multiorgan damage often seen in severe cases. Hence, there may be multiple potential mechanisms to target before irreversible end-organ dysfunction and death become the inevitable outcomes of COVID-19.

### 5.1. Colchicine

Colchicine is one of the oldest available therapies for acute gout. This drug displays anti-inflammatory effects relying on its ability to inhibit the polymerization of microtubules, and, possibly also on its action on cellular adhesion molecules and inflammatory chemokines. In addition, colchicine exerts direct anti-inflammatory and immunomodulatory effects by inhibiting the NOD-, LRR- and pyrin domain-containing protein 3 (NLRP3) inflammasome and the subsequent formation of IL-1β and IL-18 [130]. Based on these pharmacological properties, colchicine administration was identified as a possible therapy to mitigate the cytokine storm and limit multiorgan damage in severely affected COVID-19 patients. Data so far available are conflicting. A large retrospective study of 14,520 patients based on a healthcare computerized database found no significant difference in the rates of colchicine use between patients with a positive RT-PCR result for SARS-CoV-2 and those with a negative result (0.53% vs. 0.48%) [131,132]. Cure M. and colleagues has proposed an interesting speculation that would explain colchicine ineffectiveness in this setting [133]. First, we need to consider that SARS-CoV-2 entry through ACE2 is increased at a low intracellular pH. Colchicine binds microtubules in acidic condition (pH 6.7-6.8). Due to its action on microtubules, colchicine may affect pH regulation through an indirect effect on the Na^+^/H^+^ exchanger. In principle, colchicine induces a decrease of the intracellular pH, but after that, it induces a net pH increase, which may negatively impact its ability to bind microtubules. This event gives rise to a vicious cycle that prevents a further intracellular alkalinization, and, consequently, the possibility to inhibit virus binding to ACE2. Additionally, it has been proposed that colchicine could be even harmful [133] at both therapeutic and toxic doses. In the first case, colchicine may decrease the release of surfactants by affecting alveolar type II pneumocytes, whereas, at higher doses, colchicine may further inhibit surfactant secretion, worsen ARDS, and cause multiorgan failure [132,133]. Interactions with other drugs used in COVID-19 should also be considered. Colchicine metabolism relies on CYP3A4, therefore a dose adjustment could be required when drugs affecting CYP3A4 activity (i.e., macrolides or lopinavir/ritonavir) are simultaneously administered [134]. On the other hand, a single-center propensity score matched cohort study, including all consecutive COVID-19 patients admitted to a community hospital between 1 March 2020 and 30 May 2020, reports different results [135]. The primary endpoint of the study was defined as in-hospital death within 28-days follow-up. The study included 66 patients in the 1:1 matched cohort study. At the end of the 28 day follow-up, patients receiving colchicine were approximately five times more likely to be discharged and, when comparing mortality, there were 3 deaths (9.1%) in patients receiving colchicine versus 11 deaths (33.3%) in the groups receiving standard of care [135]. Findings obtained from the clinical trial GRECCO-19 (NCT04326790) [136] suggest a significant clinical benefit from colchicine in patients hospitalized with COVID-19. In this prospective, open-label, randomized clinical trial, 105 patients were randomized in a 1:1 allocation to either standard medical treatment or colchicine. Patients who received colchicine had statistically significantly improved time to clinical deterioration, without significant differences in high-sensitivity cardiac troponin or C-reactive protein levels. Although interesting, these results should be interpreted with caution. Other clinical trials evaluating the efficacy and safety of the colchicine are ongoing. COLCORONA is a randomized, double-blind, placebo-controlled trial that is evaluating the efficacy and safety of colchicine in adult COVID-19 patients who have at least one high-risk criterion (NCT04322682). The trial aims to enroll 6000 newly diagnosed patients and its primary objective is to determine whether short-term treatment with colchicine would be able to reduce the rate of death and lung complications related to COVID-19, 30 days after the enrollment. Patients will receive colchicine 0.5 mg twice daily for the first 3 days and then once daily for the remaining 27 days. The study started on March 23 and its estimated completion date is December 2020. Other clinical trials are underway (COL-COVID, NCT04350320; ColCOVID-19, NCT04322565). In consideration of the hypothesized negative effects of colchicine in terms of efficacy and safety, it will be important to further investigate its use in larger controlled trials. Findings that will emerge from ongoing clinical trials will be helpful to depict a clearer picture of the potential benefits deriving from colchicine therapy in this setting.

### 5.2. Chloroquine and Hydroxychloroquine

**Figure 4:**
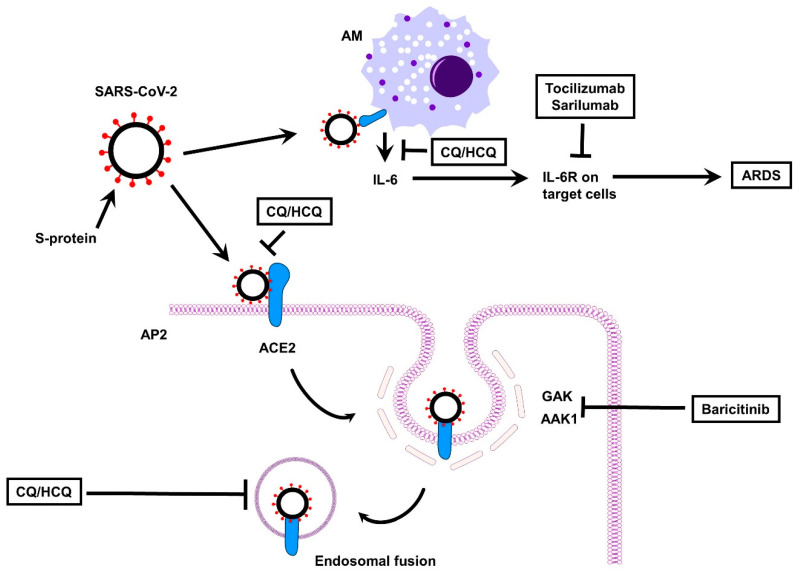
Effect of anti-rheumatic drugs on SARS-CoV-2. ACE, angiotensin-converting enzyme; AM, alveolar macrophage; AP2, alveolar pneumocyte type 2; ARDS, acute respiratory distress syndrome; CQ/HCQ, chloroquine/hydroxychloroquine; IL-6R, interleukin 6 receptor; AAK1, AP2-associated protein kinase 1; GAK, G-associated kinase; SARS-CoV-2, Severe Acute Respiratory Syndrome Coronavirus 2.

Chloroquine (CQ), an amine acidotropic form of quinine, and its more soluble and less toxic metabolite hydroxychloroquine (HCQ) have been widely used across the world for decades as front-line antimalarial agents [137]. They also have wide applications in autoimmune diseases, such as RA, juvenile idiopathic arthritis and systemic lupus erythematosus because of immunomodulatory and anti-inflammatory properties that derives from multiple mechanisms [137]. In particular, CQ and HCQ are weak bases that can be quickly concentrated in intracellular lysosome and endosomes, thereby raising the intraluminal pH of these organelles so that the processing of endogenous and exogenous ligands through lysosomes and endosomes is inhibited, the antigen presentation for the major histocompatibility complex–T cell receptor interactions is downregulated, and ultimately the downstream activation of cellular immunity is decreased [138]. Together with the ability of inhibiting NF-kB-dependent pathways, the overall effect of CQ and HCQ is to decrease production and release of various pro-inflammatory cytokines, including TNF, IL-1, IL- 6, and interferon-α (IFN-α), the major mediators of the cytokine storm syndrome. There is also evidence for anti-viral activities of CQ/HCQ against a wide range of viruses (including HIV, Ebola virus and SARS-CoV), which may synergize with their immunomodulatory and anti-inflammatory effects (Figure 4). As reported above, these lysosomotropic drugs, once entered endosomes and lysosomes, increase their pH so that the virus fusion process within the host cell and replication can be prevented [138]. In addition, both CQ and HCQ are known to interfere with the glycosylation of ACE2, which would render viral spike protein-ACE2 binding less efficient and the entry of the virus less likely [138]. These pleiotropic actions of CQ/HCQ were the rational basis for investigating their potential either in prophylaxis in high-risk population for SARS-CoV-2 infection or in the treatment of COVID-19 patients [137,139]. Although early promises about improvements in SARS-CoV-2 pneumonia, reduction in mortality and in the length of hospital stay, some later studies provided no strong evidence supporting an effective use of CQ and HCQ against COVID-19 [138,140,141,142,143,144,145]. Overall, there are still many open controversies about the potential of CQ/HCQ in the prophylaxis and therapy of COVID-19, which should be appropriately addressed in the “clean realm of science” [137,146,147]. Other issues concern the safety of CQ and HCQ. Even if these drugs have no immunosuppressant activity and in general possess a favorable safety profile, their use can be associated with several side effects, such as gastrointestinal and hepatic complications, and the most concerning ones are cardiotoxicity, myopathy and retinopathy [138]. Among these latter, cardiac toxicity deserves particular attention. CQ and HCQ are known to prolong the QT and heart rate corrected QT (QTc) interval so that patients would be exposed to the risk of serious heart disorders including arrhythmias and cardiac arrest [138,148]. In this regard, cautionary tales are repeatedly provided because COVID-19 patients may already have a prolonged QTc interval (even without CQ/HCQ use), which may be triggered by SARS-CoV-2 infection, or be linked to concurrent comorbidities and/or medications prescribed, or even traced back to predisposing genetic factors [149,150]. Daily electrocardiographic monitoring and other risk mitigation strategies (including combined assessment of baseline comorbidities and of genotype characteristics that are known pro-arrhythmic risk factors) can be readily used for eligible patients to avoid possible harms from what is currently an experimental therapy for COVID-19 patients [149]. In fact, on 15 June 2020 the FDA revoked the Emergency Use Authorization for CQ and HCQ [151], and EMA advised patients and healthcare professionals to only use CQ and HCQ for their authorized indications (including the treatment of malaria and certain autoimmune diseases), or as part of clinical trials for the treatment or prophylaxis of COVID-19, or in national emergency use programs in hospitalized patients under close supervision [152].

## 6. Mast Cell Stabilizers

Mast cells (MCs) are ubiquitous sentinels of the innate ad adaptive immunity, playing a primary role in inflammatory and allergic reactions when activated by pathogens, including viruses and bacteria that are entering into the body [49,153,154]. In particular, once activated by SARS-CoV-2, MCs located in the submucosa of the respiratory tract will then release protease, histamine, and many pro-inflammatory cytokines and chemokines that promote pulmonary complications typically seen in the most severe COVID-19 cases [49,153]. Based on the above, drugs that suppress MC activity (like cromolyn, ketotifen, quercetin, and luteolin) have the potential of alleviating the excessive inflammatory responses that accompany SARS-CoV-2 infection.

Cromolyn sodium, an anti-inflammatory agent used in the prophylactic treatment of asthma, mastocytosis, allergic rhinitis, and conjunctivitis, is able to prevent MC activation and release of inflammatory mediators, as well as to reduce adhesion molecule expression [49]. Interestingly, cromolyn sodium was found to be a potential inhibitor of Nsp12, a conserved protein in coronavirus, which is a vital RNA-dependent RNA polymerase for coronavirus replication [49]. Considering that cromolyn sodium functions exclusively as a prophylactic agent in the management of chronic symptoms, it is reasonable to expect that it would be more effective as a protective agent against ARDS associated with COVID-19, to be used at earlier stages of the disease rather than in progressive states. In terms of ongoing clinical trials, no current studies are being conducted to examine the utilization of cromolyn sodium in SARS-CoV-2.

## 7. Macrolides

Macrolides, such as azithromycin and clarithromycin, are antibacterial molecules that inhibit protein synthesis by binding to the 50S ribosomal unit of micro-organisms. These drugs also possess immunomodulatory and anti-inflammatory properties that seem not strictly related to their bacteriostatic activity. In particular, macrolides are known to decrease mucous secretion, downregulate pro-inflammatory cytokines but, at variance with CSs, do not cause immunosuppression; the exact mechanisms of these effects have been not fully elucidated [155,156,157]. Other effects promoted by macrolides, including inhibition of fibroblast proliferation, limitation of collagen production and reduced release of matrix protease, may also be relevant in the context of lung fibrosis that eventually occurs at late stage of COVID-19. Some macrolides may also have antiviral activity through the blockage of virus internalization into host cells [156,158]. Despite the above discussed mechanistic background for their use, current data are insufficient to draw any firm conclusions about the benefits of adjunctive macrolide therapy in COVID-19 patients. Furthermore, these antibiotics are known to prolong the QT interval and potentially increase the risk of sudden cardiac death. As for the use of CQ and HCQ, clinical evaluation should be combined with risk mitigation strategies in COVID-19 patients receiving this therapy, with the aim of producing net benefit over harm [147,159,160]. 

## 8. Targeting the Renin Angiotensin System (RAS) and the Kallikrein/Kinin System (KKS) in COVID-19 Disease: Opportunities and Concerns

As mentioned in the introduction, when SARS-CoV-2 bind to the membrane ectopeptidase ACE2, the number of active ACE2 expressed on cell surface becomes reduced because SARS-CoV-2/ACE2 complexes get internalized into the cells [22,26]. In this scenario, a loss of ACE2 function would favor lung inflammation and injury because two systems that cooperate in the regulation of fluid homeostasis, vascular tone and permeability, and inflammatory processes would be out of balance: the Renin Angiotensin System (RAS) and the Kallikrein/Kinin System (KKS) [161]. In particular, ACE2 represents an important crosslink regulator of angiotensin and BK metabolic pathways. On the one hand, an increased internalization of ACE2 would potentially result in unopposed functions of Ang II mediated by angiotensin type 1 receptor (AT1R) (which activates pro-inflammatory, vasoconstrictor, pro-oxidant, pro-thrombotic, and pro-fibrotic signaling pathways) because the reduced levels of Ang (1-7) and of Ang (1-9) would decrease activation of Mas receptors (MasR) and of AT2R, which instead play a key counter-regulatory role (like anti-proliferative, anti-thrombotic and anti-inflammatory) in many AT1R-related physiopathological activities [21,27]. On the other hand, following the reduction of ACE2 the levels of both BK and des-Arg9-BK can increase: in the first case, Ang II peptides that are spared from ACE2-dependent metabolism would, through a negative feedback loop, promote a secondary reduction of ACE activity, with consequent increase of BK; in the second case, loss of ACE2 would also increase the levels of des-Arg9-BK [27]. The increase in BK and des-Arg9-BK will therefore provoke an excessive activation of BK receptors (in particular B1R), and promote the so-called “bradykinin storm” [161], which would contribute to massive vascular permeability, marked rise of pro-inflammatory cytokine and ultimately incite lung inflammation and injury [26,27]. The bradykinin storm is an attractive framework to model the pathological basis for most of the characteristic COVID-19 symptoms and, similarly, offers potential targets that may have therapeutic value in clinical practice [161]. Available drugs used for the management of patients with hereditary angioedema, such as icatibant (a B2R blocker), ecallantide (a small inhibitor of plasma kallikrein), and lanadelumab (a fully human, monoclonal antibody that inhibits active plasma kallikrein proteolytic activity), may be promising candidates that require appropriate evaluation in COVID-19 patients [26,27,161,162,163,164]. A therapeutic approach based on Ang 1-7 or on Ang 1-9 would be instead problematic because of the short half-life of these peptides, whereas there are no approved drugs with equivalent pharmacodynamic profile that can be readily tested [21].

The use of drugs like ACE inhibitors (ACEIs) or Ang II receptor blockers (ARBs), which are first-choice therapeutic options for patients with hypertension, heart failure, and chronic kidney disease, has been repeatedly debated because interventions that target RAS cascade have been similarly predicted either as beneficial or harmful. Peer-review data that are currently available indicate that ACEI and/or ARB use do not increase susceptibility to SARS-CoV-2 infection, are not associated with a worse clinical outcome, and do not modify the mortality risk in COVID-19 patients [165,166,167,168,169]. Although this issue calls for more evidence, many recommendations from international regulatory agencies and scientific societies currently advise that patients on these therapies should be continued as clinically indicated, considering the potential harm that may occur with withdrawing of ACEIs or ARBs in patients with cardiovascular and other diseases [21].

## 9. Conclusions

Many countries across the world are now experiencing the long-predicted second wave of the SARS-CoV-2 pandemic that is further ravaging our society in terms of morbidities, mortalities, and economic crises. Knowledge from previous experience with SARS or MERS was not enough to limit the severity of COVID-19, and a huge array of therapies is currently tested under the urgent need to curb COVID-19 pandemic and save lives. Other than bamlanivimab, an antispike neutralizing monoclonal antibody that has been recently approved by the FDA for emergency use on COVID-19 patients during the first stages of the infection [170], vaccines are the few other medications specifically developed to halt the current pandemic that are expected to come onto the market soon. As compared to the early days of 2020, new evidences are now inspiring therapeutic strategies potentially more tailored to disease severity, but we need sound and solid supports from ongoing trials to improve the clinical practice. In this direction, many drug candidates already on the shelf are under clinical evaluation based on their ability to target SARS-CoV-2 or to quench detrimental inflammatory responses. The mainstream indication from the interim clinical experience is that anti-viral and anti-inflammatory drugs should be considered as complementary approaches that need to be timely introduced, and eventually integrated, according to COVID-19 severity: the greatest benefits for drugs that target SARS-CoV-2 entry or replication are expected at the early stage of infection, whereas anti-inflammatory drugs would be more effective in preventing/limiting the pro-inflammatory/immunopathological causes that drive the worsening of the disease. According to this before/after framework, there is minimal support (if not for harms) for a late use of anti-viral drugs, as well as for early or even prophylactic treatments with systemic anti-inflammatory drugs.
